# Design of Single-Mode Single-Polarization Large-Mode-Area Multicore Fibers

**DOI:** 10.3390/mi14101901

**Published:** 2023-10-04

**Authors:** Kamyar Rashidi, Davood Fathi, Javad Maleki, Hussein Taleb, Seyed Mohammad Mirjalili, Derek Abbott

**Affiliations:** 1 Department of Electrical and Computer Engineering, Tarbiat Modares University (TMU), Tehran 1411713116, Iran; 2 Department of Engineering Physics, Polytechnique Montréal, Montréal, QC H3C 3A7, Canada; 3 School of Electrical and Electronic Engineering, University of Adelaide, Adelaide 5001, Australia

**Keywords:** nulticore fiber, large-mode-area, single-mode, single-polarization, design, quality factor

## Abstract

In laser science and industry, considerable effort is directed toward designing fibers for fiber laser and fiber amplifier applications, each of which offers a particular advantage over the others. Evanescently coupled multicore fibers, however, have been studied less extensively due to the relatively small mode area in the single-mode regime. Here, by proposing a new structure with stress-applying parts in a 37-core fiber and optimizing this structure through a comprehensive framework, we present 21 solutions characterized by large-mode-area and high beam quality in the single-mode, single-polarization regime. Different fiber designs are optimal for different output parameters. In one design, the mode area can significantly increase to above 880 μm${}^{2}$, which is comparable with that of photonic-crystal fibers. Moreover, besides the single-mode operation, the beam quality factor ($M^{2}$ factor) of the fundamental mode is considered an output parameter in the bent state and is improved up to 1.05 in another design. A comprehensive tolerance analysis is then performed to assess the performance of the designs under deviations from normal conditions. Moreover, in spite of the shifts in the loss of modes, the proposed high beam quality LMA fibers maintain single-polarization, single-mode operation across a wide range of core pitches, bending orientation angles, and bending radius deviations. Our results highlight the potential of multicore fibers for the efficient operation of fiber lasers and amplifiers.

## 1. Introduction

High-power fiber lasers have been used as alternatives for semiconductor lasers and other related technologies due to their high output power, beam quality, efficient heat dissipation, and compactness [1,2,3,4,5,6]. These types of fibers are also used in sensors. One of the prominent advantages of fiber sensors based on photonic-crystal is their easy manufacturing compared to other sensors [7,8,9,10]. The most important characteristic of fibers designed for fiber lasers and fiber amplifiers is LMA (Table 1 indicates the acronyms.), which is essential for increasing the delivered power, reducing nonlinear effects, and ensuring single-mode operation to enhance beam quality and mode stability. In addition, to achieve compact and low-footprint devices, the fiber is coiled, necessitating that the bending loss of the FM be minimized as much as possible [1,2,11,12,13]. Furthermore, to maintain a simple laser setup and to avoid a reduction in the degree of polarization, the use of single-polarization fibers, or polarization-preserving fibers, is necessary [14,15,16,17,18].

Increasing the EMA in conventional fibers by increasing the core diameter can severely degrade the beam quality and mode stability and lead to the excitation of HOMs. Moreover, bending the fiber highly increases FM loss in conventional fibers. Regarding these issues, there is an inherent trade-off between the conflicting FOMs of the fiber [19]. Several different structures, such as LMA fibers with asymmetric bend compensation, segmented cladding fibers, PCFs, LCFs, and MCFs, have been proposed to reduce the trade-off between the mode area, single-mode operation, and FM loss of bent fibers [20,21,22,23,24,25,26,27,28,29,30]. Additionally, in polarization-preserving fibers, the birefringence induced by stress using SAPs not only removes one of the polarizations of all modes but also decreases the FM-guided polarization leakage [15]. Another difficulty that arises as a result of both bending the fiber and applying birefringence is the deformation of the Gaussian profile of the FM. Although single-mode operation—achieved by increasing HOM loss and decreasing FM loss—results in high output beam quality and mode stability, the deformation of the FM as a result of fiber bending makes beam focusing more challenging [1,2,16,17,31,32]. Among all the proposed structures, multicore fibers present another suitable option, offering diffraction-limited output beam quality, a less complicated cladding structure compared to PCFs and LCFs, and a higher numerical aperture than step-index fibers [20,33]. The air holes in the cladding of a PCF must be precisely spaced and aligned. Even small imperfections can lead to additional losses [34]. However, the EMA of all-solid multicore fibers is low in the SM-operating regime [20,33,35]. It would be rational to think that by combining the advantages of multicore fibers with single-polarization fibers, one can improve the characteristics of the fiber. However, the conflict between output parameters increases due to the complicated nature of the fiber and the interplay between different physical parameters. To strike a balance between EMA, single-mode and single-polarization operation, and high beam quality, artificial intelligence techniques can be utilized [36,37,38,39,40,41], among which, the framework proposed in [42] is more compatible with our work. Furthermore, all solid dual-core fibers and dual-core photonic-crystal fibers have been designed and optimized to enhance the large-mode-area characteristic in single-mode operation [43].

In this paper, we design single-mode, single-polarization, large-mode-area multicore fibers (SM-SP-LMA-MCFs) that yield diffraction-limited output beam quality. In addition to utilizing 37 coupled cores to ensure a large mode area, SAPs are embedded on both sides of the core region to induce birefringence, thereby eliminating one of the polarizations. For the first time, to the best of our knowledge, apart from improving SM operation to improve beam quality, the $M^{2}$ factor of the FM in the bent state is introduced as an output parameter to the simulations to prevent the deformation of the FM in the coiled fiber. Then, considering all figures of merit and using a comprehensive multi-objective optimization framework, the fiber is optimized, and the results are analyzed with regard to the effects of FOMs. This paper is organized as follows. Section 2 describes the proposed design, the method of analysis, and the optimization framework. Section 3 presents the results, and the sensitivity of the optimal structures to variations in different structural parameters is meticulously investigated. Finally, the paper is summarized in Section 4.

## 2. Structure and Theory

### 2.1. Designof the Single-Mode Single-Polarization Large-Mode-Area Multicore Fiber

**Figure 1:**
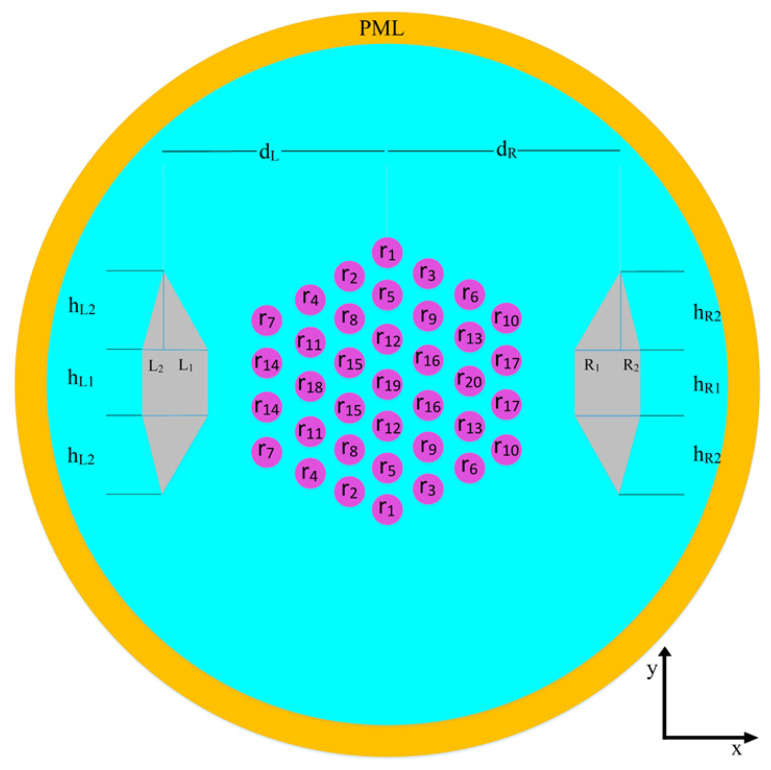
The transverse cross-section of the proposed SM-SP-LMA-MCF.

Figure 1 shows the two-dimensional schematic of the proposed SM-SP-LMA-MF with diffraction-limited output beam quality. To remove one polarization of the fundamental mode in a high beam quality fiber, boron-doped silica, as SAPs, have been used on both sides in a multicore fiber [15]. Instead of using a 19-core fiber proposed in [20], 37 cores are arranged in a hexagonal lattice to ensure a larger mode area. As illustrated in Figure 1, parameters $r_{1}$ to $r_{20}$ represent the radii of cores, Λ indicates the core pitch, $d_{L}$ and $d_{R}$ denote the distance of the left and right SAPs, and parameters $h_{L1}$, $h_{L2}$, $l_{L1}$, $L_{L2}$, $h_{R1}$, $h_{R2}$, $l_{R1}$, and $l_{R2}$ represent the dimensions of the left and right SAPs. Since the exerted bending is considered to be only along the x-direction, the structure is designed to be symmetrical along the y-direction. The background material is silica with a refractive index of 1.4496, and the NA of each core is 1.08 at Λ = 1.064 μm [20,42]. The boron-doped silica used in SAPs has a refractive index [15] of 1.4416. Since the core region can be co-doped with materials, such as fluorine and boron oxide (to have a refractive index equal to the refractive index of pure silica), the results are valid for both active and passive fibers [13]. More information about the thermal expansion coefficient, Young’s modulus, Poisson’s ratio, and photoelastic constant can be found in reference [44].

### 2.2. Theoretical Description

The mode calculation is implemented in two steps. In the first step, the refractive index change induced by SAPs is calculated, whereas, during the second step, the calculated refractive index tensor is used to solve the Helmholtz equation via FEM in association with the PML boundary condition, thereby calculating the eigenvalues and the mode profiles.

In the first step, the new refractive index profile induced by thermal expansion is evaluated. Using the following equilibrium equation (Equation (1)), the stresses induced by thermal expansion can be calculated using [44]

(1) $$\nabla .\sigma =-\nabla .\left(\begin{array}{c} \left[\begin{array}{c} \varepsilon_{x}\\ \varepsilon_{y}\\ \gamma_{xy} \end{array}\right]-\left[\begin{array}{c} \alpha \\ \alpha \\ 0 \end{array}\right]\left(1+\upsilon \right)\left(T-T_{\mathrm{ref}}\right) \end{array}\right),$$

(2) σ=Dε,

where $T_{ref}$ denotes the reference temperature, *T* is the annealing temperature, υ is Poisson’s ratio, α represents the expansion coefficient, $\varepsilon_{x(y)}$ and $\gamma_{xy}$ are the strain components, σ is the stress tensor, and *D* is the elasticity matrix. The relationship between the applied stress and the change in refractive index (Δ*n*) can be calculated according to the elasto-optical effect equation [44]:

(3) Δn=−Cσ.

Using Equation (2) and with the values of $C_{1}$ and $C_{2}$ given in references [15,44], the birefringence (*B*) is calculated using the following equation:

(4) $$B=|\left(C_{2}-C_{1}\right)\left(\sigma_{x}-\sigma_{y}\right)|.$$

In the second step, using the refractive index tensor calculated in the previous step, we solve the vector Helmholtz equation [45]:

(5) $$\Delta \times \left(\Delta \times E\right)-k_{0}^{2}n^{2}E=0,$$

where *E* represents the electric field vector, $k_{0}$ is the wavenumber in the free space, and *n* is the modified refractive index. The output parameters that are required to evaluate the performance of the designed fiber are the effective mode area, bending loss, and $M_{2}$ factor. The bending loss of each mode (dB/m) is calculated using the imaginary part of its effective refractive index [27]:

(6) $$L=8.686k_{0}Im\left(n_{eff}\right),$$

where $n_{eff}$ is the effective index of the mode, and $k_{0}$ is the free space wavenumber (1/m). The EMA of the propagating mode can be calculated using [27]:

(7) $$A_{eff}=\frac{{\left({\int \int |E|}^{2}dxdy\right)}^{2}}{{\int \int |E|}^{4}dxdy}.$$

The $M^{2}$ factor of a propagating mode is calculated according to $M_{x}^{2}$ and $M_{y}^{2}$ using the following equations proposed in [46],

(8) $$M^{2}=\sqrt{M_{x}^{2}M_{y}^{2}},$$

where

(9) $$M_{j}^{2}=4\pi \sigma_{0j}^{2}\sigma_{\infty j}^{2}\;\mathrm{For}\;j\;=\;x\;\mathrm{or}\;y.$$

Let j = x or y, and terms $\sigma_{0j}$ and $\sigma_{\infty j}$ are the second-order moments in the spatial and frequency domains, respectively. Term $\sigma_{0j}$ can be calculated using [46]

(10) $$\sigma_{0j}^{2}=\frac{\int_{-\infty }^{\infty }\int_{-\infty }^{\infty }{\left(j-\bar{j}\right)}^{2}{\left|E\left(x,y\right)\right|}^{2}dxdy}{\int_{-\infty }^{\infty }\int_{-\infty }^{\infty }{\left|E\left(x,y\right)\right|}^{2}dxdy},$$

where the first-order moment in the spatial domain of a given mode is given by [46]

(11) $$\bar{j}=\frac{\int_{-\infty }^{\infty }\int_{-\infty }^{\infty }j{\left|E\left(x,y\right)\right|}^{2}dxdy}{\int_{-\infty }^{\infty }\int_{-\infty }^{\infty }{\left|E\left(x,y\right)\right|}^{2}dxdy}.$$

Based on the 2D-Fourier transform theory, the field distribution in the frequency domain *E*($f_{x},f_{y}$) is obtained by [46]

(12) $$E\left(f_{x},f_{y}\right)=\int_{-\infty }^{\infty }\int_{-\infty }^{\infty }exp\left[-i2\pi \left(f_{x}x+f_{y}y\right)\right]dxdy,$$

where $f_{x}$ and $f_{y}$ are the transverse spatial frequencies. According to the frequency domain distribution of the field, the second-order moments in the frequency domain can be given by [46]

(13) $$\sigma_{\infty }^{2}=\frac{\int_{-\infty }^{\infty }\int_{-\infty }^{\infty }\left(f_{j}-{\bar{f}}_{j}\right){\left|E\left(f_{x},f_{y}\right)\right|}^{2}df_{x}df_{y}}{\int_{-\infty }^{\infty }\int_{-\infty }^{\infty }{\left|E\left(x,y\right)\right|}^{2}df_{x}df_{y}},$$

where the first-order moment in the frequency domain of a given mode is given by [46]

(14) $${\bar{f}}_{j}=\frac{\int_{-\infty }^{\infty }\int_{-\infty }^{\infty }f_{j}{\left|E\left(f_{x},f_{y}\right)\right|}^{2}df_{x}df_{y}}{\int_{-\infty }^{\infty }\int_{-\infty }^{\infty }{\left|E\left(x,y\right)\right|}^{2}df_{x}df_{y}}.$$

### 2.3. Optimization Framework

Using MOGWO in combination with an image processing technique—to classify all modes according to their order—we can optimize the proposed structure and offer designs that can provide worthy characteristics [42]. In addition to calculating the area and loss of modes using the FEM simulator, the effect of stress induced by thermal expansion on the refractive index profile and the $M^{2}$ factor have been included in the framework.

All the structural parameters, including the radii of the cores, the core pitch, SAP dimensions, and SAP distances from the core, are defined in a PM to be optimized.

(15) $$PM=[r_{1}/\Lambda ,\ldots ,r_{20}/\Lambda ,h_{L1}/\Lambda ,h_{L2}/\Lambda ,l_{L1}/\Lambda ,l_{L2}/\Lambda ,h_{R1}/\Lambda ,h_{R2}/\Lambda ,l_{R1}/\Lambda ,l_{R2}/\Lambda ,d_{L}/\Lambda ,d_{R}/\Lambda ,\Lambda ]$$

Moreover, all fabrication and bending limitations, along with the desired range for the objectives, are incorporated in a CM, see Table 2. The beam quality factor in the x and y directions can be improved by decreasing the $M^{2}$-factor and the term $|M_{x}^{2}-M_{y}^{2}|$.

**Figure 2:**
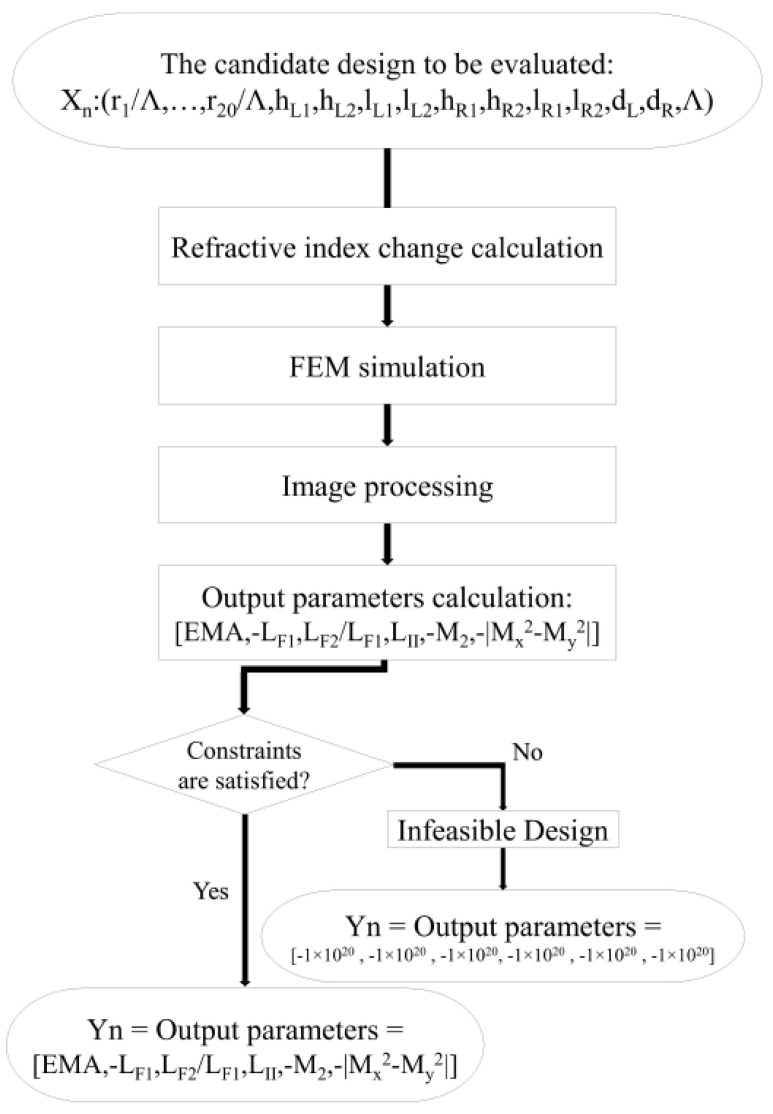
The modified module for calculating output parameters.

Figure 2 shows the calculation process of the output parameters. After calculating the refractive index change induced by SAPs for the candidate design, the FEM simulator calculates the mode profiles and their effective refractive indices. Mode order recognition is performed using image-processing techniques. Using the information from the image-processing part and FEM simulator, the EMA, $-L_{FX}$, $L_{FY}$/$L_{FX}$, $-L_{H}$, $-M^{2}$, and $-|M_{x}^{2}-M_{y}^{2}|$ are calculated. As the optimizer attempts to increase all the objectives, the objectives that should be decreased are multiplied by −1, such as $-L_{FX}$, $-M^{2}$, and $-|M_{x}^{2}-M_{y}^{2}|$. If the merit factors are not in the acceptable range defined by the CM, a very low value $\left(-1\times 1^{20}\right)$ will be assigned to them. Hence, the optimizer can find that such candidate designs are not feasible and, consequently, they are neglected during the optimization process.

## 3. Results and Discussion

The parameters of 21 optimal designs, characterized by a large mode area, low bending loss for the x-polarized FM ($LF_{X}$), high bending loss for the y-polarized FM ($LF_{Y}$), and HOMs ($L_{H}$), along with high beam quality (indicated by a low $M^{2}$ factor and $|M_{x}^{2}-M_{y}^{2}|$) are reported in Table A1, as detailed in Appendix A. Simulations are performed for a bending radius of 20 cm and a wavelength of 1064 nm. Given the many conflicting output parameters, the optimization problem has several solutions. These solutions are a set of solutions that trade-off these output parameters. All solutions have a standard output range for all parameters, and each design has at least one advantage over all other designs, and some designs may have multiple advantages. For example, design no. 9 has a higher EMA when compared to nos. 2, 3, 4, 5, 6, 7, 8, 10, 11, 12, 14, 16, 17, 18, 19, 20, and 21, as well as higher LH in comparison with nos. 1, 2, 5, 13, 14, 15, 16, and 21. While keeping other objectives in the desirable range by putting constraints on them, the EMA, $L_{FY}$/$L_{FX}$, and $L_{H}$ can be increased separately to the range of 880 (μm${}^{2}$), 4.95, and 485 dB/m for design nos. 15 and 17.

**Figure 3:**
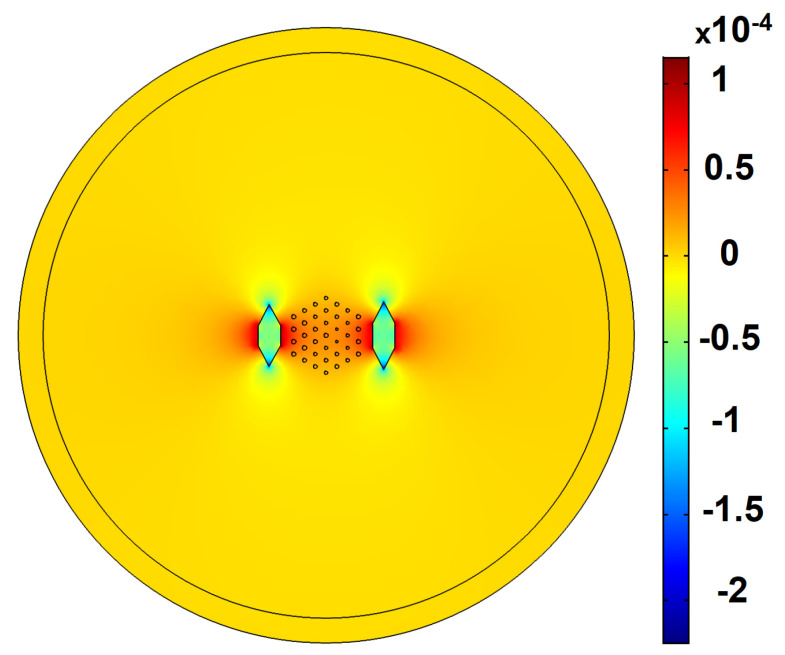
The birefringence distribution induced by SAPs in SM-SP-LMA-MCFs.

Likewise, without degrading the values of other FOMs to the undesirable regime, LFY, $M^{2}$, and $|M_{x}^{2}-M_{y}^{2}|$ are decreased to the range of 0.12 dB/m, 1.05, and 0 for design nos.19 and 5. Figure 3 illustrates the distributed birefringence induced by SAPs at the fiber cross-section. The refractive index in the y-direction ($n_{y}$) is so decreased that the effective refractive index of the y-polarized fundamental mode is in the same range as the cladding index. As a result, index-matched coupling occurs between y-polarized FM and cladding, which increases the loss of the y-polarized fundamental mode. As can be seen in Table 2, the height of SAPs is increased to the maximum range, around 2 Λ, to increase birefringence. Nevertheless, a further increase in the height of SAPs reduces the loss of all modes, including $L_{FY}$ and $L_{H}$. In fact, since the refractive index of the boron-doped silica is below the refractive index of cores and fused silica, they act as a barrier to the modes. Similarly, $d_{L}$ and $d_{R}$—which imply the distance of SAPs from the center of the fiber—are in the range of 3.2 Λ–3.82 Λ, which means that they are placed near the core region to increase birefringence. Nonetheless, decreasing the distance gives rise to a reduction in the core area and, hence, there is a decrease in EMA. The average value of the left, middle, and right core radii are 0.1573 Λ, 0.1490 Λ, and 0.1462 Λ, respectively. Indeed, to compensate for the effect of bending, the refractive index in the bending direction is decreased, which is in agreement with the results reported in the literature [13,21,26].

**Figure 4:**
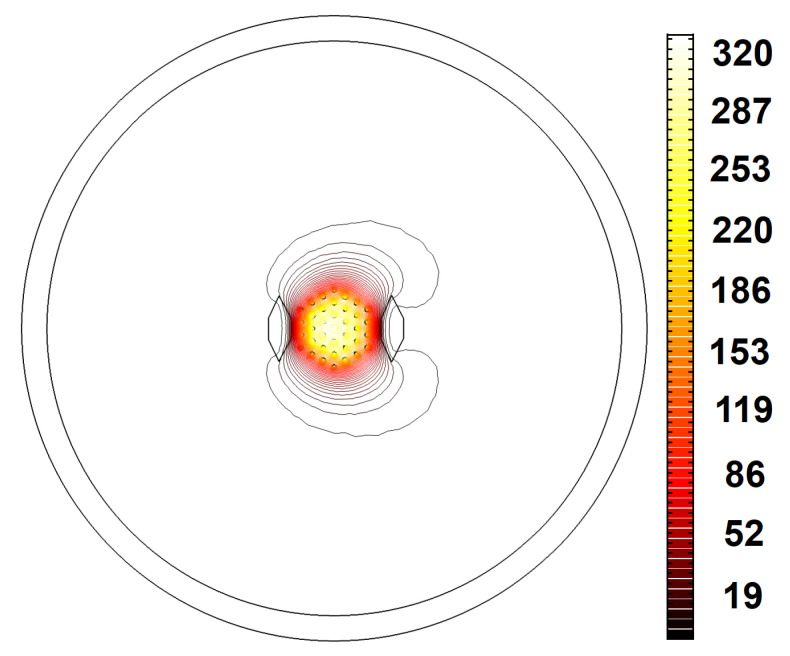
The electric field distribution (V/m) of the x-polarized fundamental mode for design no. 19.

**Figure 5:**
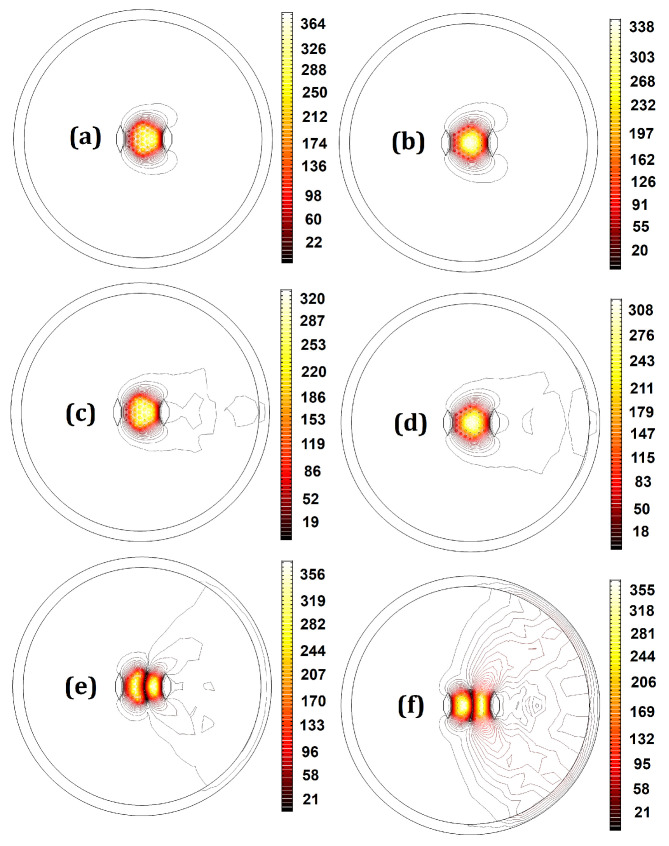
Mode field distribution for (**a**) the x-polarized FM of design no. 15, (**b**) the x-polarized FM of design no. 17, (**c**) the y-polarized FM of design no. 15, (**d**) the y-polarized FM of design no. 17, (**e**) the HOM of design no. 15, and (**f**) the HOM of design no. 17.

The designs representing the best structures with the highest EMAs, $L_{FY}$/$L_{FX}$, $L_{H}$, and $L_{FY}$, as well as the lowest, $M^{2}$, $L_{FX}$, and $|M_{x}^{2}-M_{y}^{2}|$, are highlighted in bold in Table A1 in Appendix A. Design no. 19—with the highest beam quality—has an $M^{2}$ factor of 1.05 in the bent state. Designing a fiber with an $M^{2}$ factor lower than 1.1 in the 20 cm bending radius is a problem. Despite this, a multicore fiber with a proper core arrangement and SAP dimensions can achieve a low deformation of the x-polarized fundamental mode. As can be seen in Figure 4, design no. 19 has a low bending loss and exhibits a Gaussian beam profile for the x-polarization of the fundamental mode. According to Table A1 in Appendix A, design no. 15—with the highest EMA—has the lowest acceptable $L_{H}$. This structure has the highest core pitch (5.8 μm) among all designs, so the fundamental mode occupies a larger area. Given the fact that there is a trade-off between the EMA and single-mode operation, this design has the lowest acceptable $L_{H}$. Figure 5 provides clear evidence about $L_{FX}$, $L_{FY}$, and $L_{H}$ in design nos. 15 and 17. Regarding the values of $L_{FX}$, $L_{FY}$, and $L_{H}$, we can see that the leakier the mode, the higher its loss. Due to the low leakage of the HOM power in the cladding, the $L_{H}$ of design no. 15 is the lowest among all structures. Therefore, this structure has the least effective single-mode operation. On the other hand, as can be seen in Figure 5f, the optical field strongly penetrates the cladding region and, therefore, the lower confinement level of the HOM in design no. 17 is a testament to its higher $L_{H}$ and effective single-mode operation.

Designing high-quality practical fibers requires considering all fabrication variations. Additionally, the versatility of the design enables it to operate in different settings, which is of great interest to end-users. In the following, the sensitivity of the fiber to the core pitch, the bending orientation, and the bending radius is investigated. Taking into account fiber output values in addition to sensitivities, users can select their desired design among the proposed designs.

**Figure 6:**
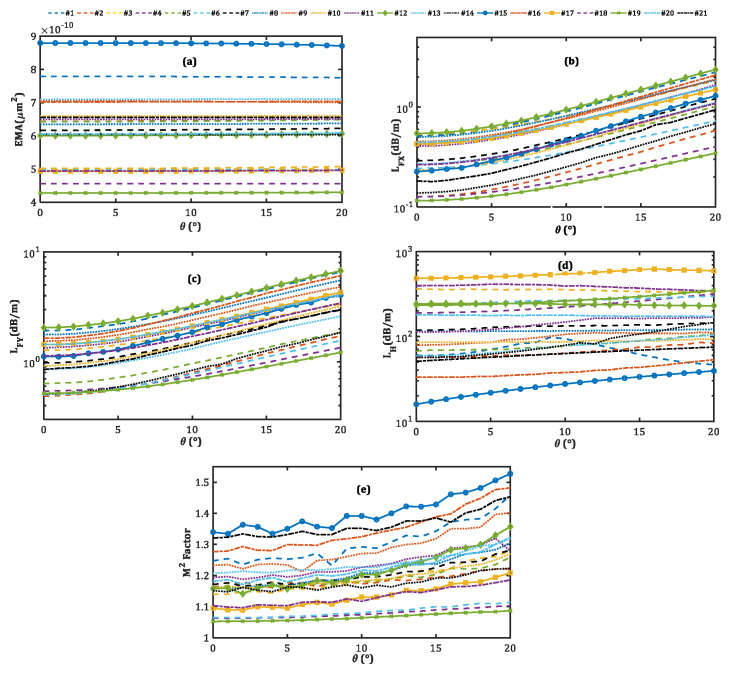
Variations of the (**a**) EMA, (**b**) $L_{FX}$, (**c**) $L_{FY}$, (**d**) $L_{H}$, and (**e**) $M^{2}$ factor on the bending orientation angle.

Because unwanted changes in the bending orientation are inevitable during the bending process, evaluating the fiber characteristics under different bending orientation angles is of great importance [31]. Having a large Gaussian-like mode profile of the x-polarized fundamental mode confined to the core region is crucial for sustaining efficiency across various bending orientation angles, in addition to preserving single-mode and single-polarized operations. Figure 6 illustrates the effect of the bending orientation angle (θ) on the FOM performances of 21 optimal designs. We note that the EMAs for all designs are kept at the same level when the bending orientation angles change. However, both $L_{FX}$ and $L_{FY}$ increase with the increase in the bending orientation angle. Because the fiber is designed to work optimally at $\theta =0^{\circ }$, the optimal refractive index profile of the fiber’s cross-section is for this condition. Thus, changes in the value of θ result in deviations in the refractive index profile. The larger the changes in the bending orientation angle, the more pronounced the modifications are in the refractive index of the fiber cross-section, leading to more leakage of both polarizations of the fundamental mode. In addition, since the SAPs—both of which have lower refractive indices than background material—are positioned in the bending direction, they act as strong barriers to the modes’ expansion into the cladding region.

**Figure 7:**
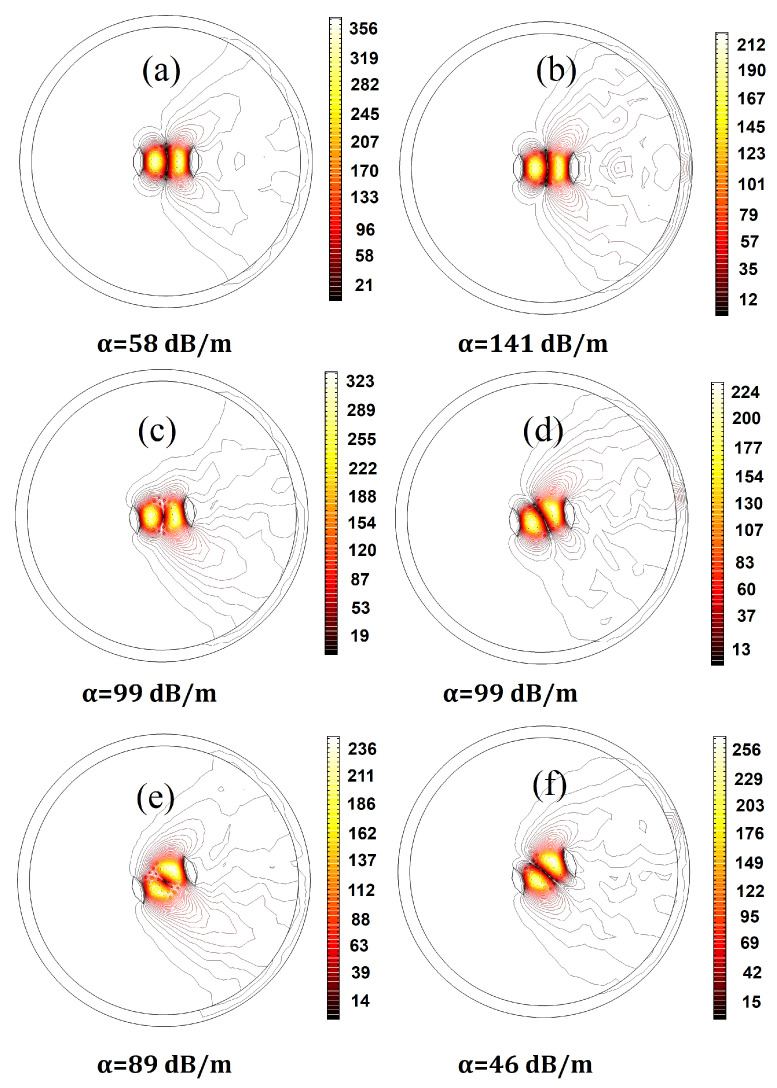
The electric field distribution of (**a**) x-polarized LP11, observed with $\theta =0^{\circ }$ (**b**) y-polarized $LP_{11}$, observed with $\theta =0^{\circ }$ (**c**) x-polarized $LP_{11}$, observed with $\theta =9^{\circ }$ (**d**) y-polarized $LP_{11}$, observed with $\theta =9^{\circ }$ (**e**) x-polarized $LP_{11}$, observed with $\theta =20^{\circ }$ (**f**) y-polarized $LP_{11}$, observed with $\theta =20^{\circ }$.

Nevertheless, by rotating the fiber along its axis, the barrier-like behaviors of SAPs in some areas of the fiber cross-section decrease and, hence, increase mode losses. In the same way, the $L_{H}$ increases as the bending orientation angle increases. However, in the case of design no. 1, it is not an upward curve, rather, it reaches a peak at $\theta =9^{\circ }$. While x-polarized $LP_{11}$ has the lowest loss among higher-order modes for $\theta =0^{\circ }$ in all designs, the loss of x-polarized $LP_{11}$ exceeds that of y-polarized $LP_{11}$, even for $\theta >9^{\circ }$ in design no. 1. Therefore, since we have introduced the $L_{H}$ as the minimum higher-order mode loss, $L_{H}$ corresponds to the loss of the y-polarized LP11, even for $\theta >9^{\circ }$. Figure 7 provides valuable information about the mode profile and bending loss of the x- and y-polarized LP11, even in the bending orientation angles of $0^{\circ }$, $9^{\circ }$, and $20^{\circ }$ for design no. 1. As the bending orientation angle increases, $n_{y}$ increases, thus decreasing the loss of the y-polarization of $LP_{11}$, which is associated with $L_{H}$ for $\theta >9^{\circ }$. Lastly, for all designs, because the mode profile of x-polarized FM slightly changes by changing the fiber orientation from a vertical position, the $M^{2}$ factor increases. For most of the designs, this parameter is in a standard range. Considering the sensitivities of all these parameters to bending orientation angle, we note that the large-mode-area fiber maintains both single-mode and single-polarized operation under varying bending orientations, at the cost of an increase in $L_{FX}$.

**Figure 8:**
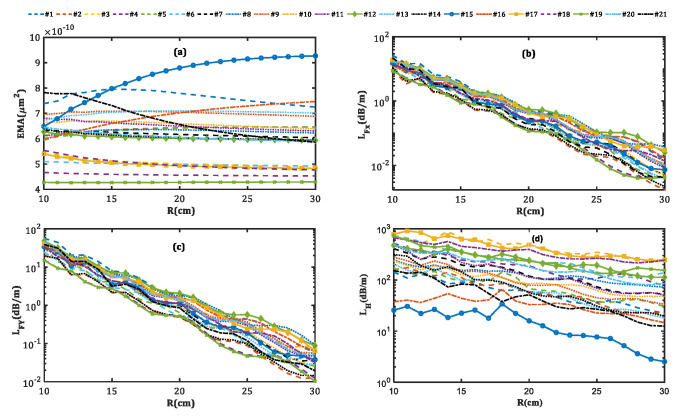
(**a**) EMA (**b**) $L_{FX}$, (**c**) $L_{FY}$, and (**d**) $L_{H}$ behavior versus the bending radius.

**Figure 9:**
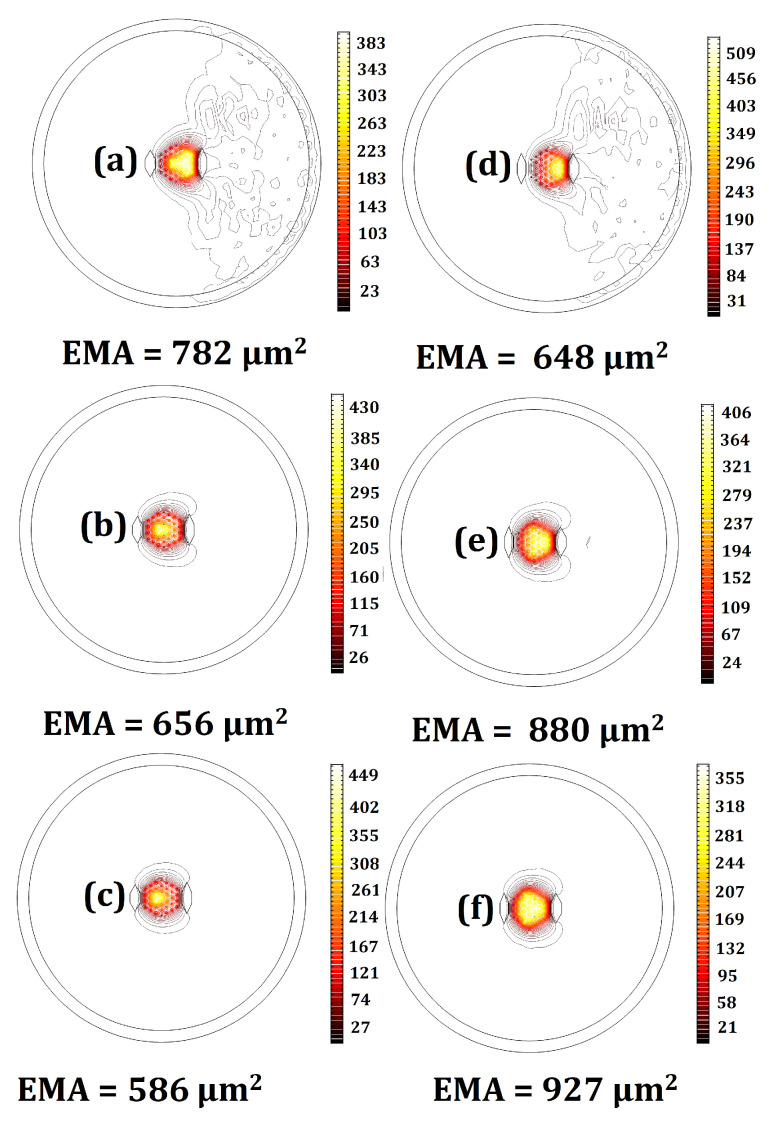
Mode field distribution for the x-polarized FM of design no. 21 with (**a**) R = 10 cm, (**b**) R = 20 cm, and (**c**) R = 30 cm, and design no. 15 with (**d**) R = 10 cm, (**e**) R = 20 cm, and (**f**) R = 30 cm.

Figure 8 shows the dependence of fiber characteristics on the bending radius. As can be seen from Figure 8a, EMA is minimally affected by the bending radius changes for the majority of designs. Among all designs, design nos. 15 and 21 with high core pitches are more sensitive to the bending radius. Despite this, the EMAs of these designs remain large even in small bending radii. The profiles of the x-polarized FMs in both designs with bending radius values of 10, 20, and 30 cm are shown in Figure 9.

The confinement increases with the bending radius in both designs, yet the localization increases in design no. 21 and decreases in design no. 15. This contrast leads to a reduction of EMA in the former and an improvement in the latter. Moreover, the smaller the fiber bending radius, the higher the $L_{FX}$, $L_{FY}$, and $L_{H}$ for all designs. Thus, in the bending radius range of 10 to 30 cm, all designs remain in the single-mode single-polarization operation, but in a different loss range. For small bending radii, $L_{FX}$ is weakly guided but its difference with the y-polarization of the fundamental mode loss and HOMs is so high that the single-mode single-polarization operation is assured. On the other hand, as can be seen in Figure 8b,c, for large bending radii, where $L_{FX}$ and $L_{FY}$ are low, the loss of the x-polarized fundamental mode is low enough to obtain the single-mode single-polarization operation.

**Figure 10:**
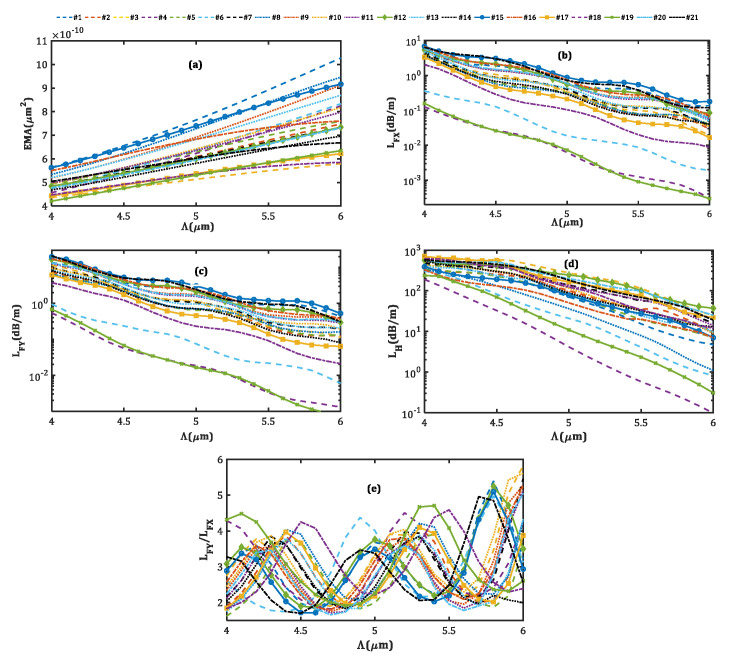
The effect of the core pitch size on the (**a**) EMA, (**b**) $L_{FX}$, (**c**) $L_{FY}$, (**d**) $L_{H}$, and (**e**) $L_{FY}/L_{FX}$.

Figure 10 indicates the dependence of output parameters on the core pitch (Λ). In this case, since the value of all structural parameters is defined as a ratio to the pitch, increasing the core pitch leads to an increase in the SM SP LMA MCF scale as a whole. As a result, the larger the core pitch, the larger the area the modes occupy, and the greater the EMA (Figure 10a). However, due to the weak coupling between the modes in two adjacent cores and larger core sizes in the larger pitch, the bending loss for all modes decreases as well, as seen in Figure 10b–d. This result is in agreement with the step-index fibers, where an increase in the core size results in the decreasing mode loss and appearance of HOMs. In addition to changing the refractive index, doping the fiber with rare-earth dopants can also change the spectroscopic properties of the core medium, such as the absorption and fluorescence spectra [47]. Furthermore, the quantum defect caused by the rare-earth dopants in the core medium results in significant heating of the core during high-power operations [48].

## 4. Conclusions

In summary, we proposed a novel large-mode-area fiber, implementing stress-applying parts in the cladding region of a multicore fiber. This proposed structure was optimized using a multi-objective optimization technique, considering a bending radius of 20 cm and a wavelength of 1.064 μm. Accordingly, 21 optimal designs were obtained, each demonstrating single-mode, single-polarization operation, large mode area, and high beam quality. Different designs offer trade-offs between various output parameters. We demonstrated that one of the optimal designs represents the highest recorded effective mode area of 880 μm${}^{2}$. An x-polarized fundamental mode loss of 0.12 dB/m, a y-polarized FM loss of 2.05, and a higher-order mode loss of 485 dB/m are among the best-recorded merit factors of our optimal designs. Apart from single-mode operation, a beam quality factor of 1.05 guarantees the fiber’s beam quality in the bent state. Additionally, the sensitivity of all designs to the core pitch size, bending radius, and bending orientation angle were studied in detail. We find that the large effective mode area of the fiber can be guaranteed, even under different bending radii and bending orientation angles. Moreover, despite the changes in the loss of the x-polarized fundamental mode, it was observed that the fiber maintains single-mode and single-polarization operations under different core pitch sizes, bending orientation angles, and bending radii.

## Figures and Tables

**Table 1:** Acronyms.

Acronyms	Meaning	Acronyms	Meaning
SAPs	Stress-applying parts	LMA	Large-mode-area
FM	Fundamental mode	EMA	Effective Mode Area
HOMs	Higher-order modes	FOMs	Figure of merits
PCFs	Photonic-crystal fibers	LCFs	Leaky channel fibers
MCFs	Multicore fibers	SM	Single-mode
SP	Single-polarization	NA	Numerical aperture
FEM	Finite element method	PML	Perfectly matched layer
EMA	Effective mode area	CM	Constraint module
MOGWO	Multi-objective gray wolf optimizer	PM	Parameter module

**Table 2:** Range of input and output Parameters.

Parameters	Min	Max
Λ (μm)	4	10
$\left[r_{1},r_{2},\ldots ,r_{19},r_{20}\right]$	0.1 Λ	0.4 Λ
$\left[h_{l1},h_{l2},h_{r1},h_{r2}\right]$	0	2 Λ
$\left[l_{l1},l_{l2},l_{r1},l_{r2}\right]$	0	1 Λ
$d_{1}$	−5 Λ	−3.2 Λ
$d_{2}$	3.2 Λ	5 Λ
EMA (μm${}^{2}$)	400	-
$L_{FX}$ (dB/m)	-	0.6
$L_{FY}/L_{FX}$	2	-
$L_{H}$ (dB/m)	15	-
$L_{FY}$ (dB/m)	0.5	-
$M^{2}$	1	1.4
$|M_{x}^{2}-M_{y}^{2}|\;\times \;10^{3}$	0	0.2

## Data Availability

The datasets used and/or analyzed during the current study are available upon reasonable request from the corresponding author.

## Appendix A

**Table A1:** Structural parameters of the 21 SM-SP-LMA-MCF design.

	#1	#2	#3	#4	#5	#6	#7	#8	#9	#10	#11	#12	#13	#14	#15	#16	#17	#18	#19	#20	#21
$r_{1}/\Lambda$	0.13	0.13	0.11	0.18	0.16	0.15	0.14	0.15	0.15	0.15	0.13	0.11	0.15	0.11	0.14	0.17	0.1	0.13	0.15	0.13	0.1
$r_{2}/\Lambda$	0.15	0.18	0.16	0.16	0.15	0.17	0.18	0.12	0.15	0.14	0.14	0.14	0.18	0.17	0.19	0.18	0.17	0.15	0.18	0.16	0.19
$r_{3}/\Lambda$	0.16	0.17	0.16	0.21	0.18	0.18	0.17	0.18	0.19	0.17	0.19	0.17	0.19	0.18	0.15	0.19	0.17	0.18	0.21	0.14	0.14
$r_{4}/\Lambda$	0.15	0.14	0.11	0.15	0.14	0.16	0.14	0.15	0.12	0.14	0.15	0.12	0.16	0.14	0.12	0.12	0.12	0.13	0.14	0.13	0.14
$r_{5}/\Lambda$	0.15	0.15	0.1	0.19	0.14	0.18	0.13	0.15	0.14	0.14	0.14	0.12	0.15	0.16	0.17	0.13	0.14	0.11	0.16	0.12	0.11
$r_{6}/\Lambda$	0.14	0.15	0.13	0.14	0.13	0.13	0.16	0.16	0.17	0.14	0.15	0.13	0.13	0.12	0.14	0.16	0.15	0.14	0.16	0.16	0.15
$r_{7}/\Lambda$	0.17	0.15	0.14	0.15	0.13	0.13	0.16	0.14	0.14	0.15	0.16	0.17	0.14	0.18	0.1	0.12	0.13	0.19	0.16	0.15	0.16
$r_{8}/\Lambda$	0.14	0.13	0.13	0.18	0.16	0.17	0.12	0.17	0.13	0.14	0.11	0.13	0.12	0.12	0.16	0.11	0.15	0.12	0.13	0.14	0.11
$r_{9}/\Lambda$	0.14	0.12	0.12	0.17	0.12	0.14	0.12	0.11	0.14	0.14	0.13	0.16	0.11	0.16	0.11	0.15	0.13	0.14	0.16	0.13	0.11
$r_{10}/\Lambda$	0.17	0.17	0.12	0.15	0.18	0.13	0.16	0.17	0.16	0.16	0.13	0.13	0.17	0.12	0.15	0.18	0.13	0.17	0.15	0.17	0.17
$r_{11}/\Lambda$	0.18	0.18	0.2	0.2	0.18	0.2	0.17	0.21	0.17	0.17	0.18	0.15	0.19	0.14	0.18	0.15	0.18	0.17	0.18	0.15	0.15
$r_{12}/\Lambda$	0.13	0.15	0.16	0.18	0.16	0.17	0.14	0.15	0.16	0.15	0.16	0.17	0.13	0.16	0.14	0.14	0.17	0.19	0.22	0.18	0.12
$r_{13}/\Lambda$	0.12	0.15	0.13	0.19	0.13	0.18	0.14	0.15	0.14	0.14	0.15	0.13	0.17	0.17	0.14	0.16	0.13	0.13	0.16	0.15	0.12
$r_{14}/\Lambda$	0.18	0.15	0.13	0.14	0.16	0.15	0.12	0.16	0.14	0.15	0.13	0.13	0.12	0.12	0.12	0.16	0.16	0.12	0.14	0.15	0.16
$r_{15}/\Lambda$	0.16	0.18	0.18	0.19	0.17	0.17	0.19	0.16	0.18	0.19	0.19	0.18	0.18	0.18	0.14	0.17	0.19	0.2	0.17	0.18	0.2
$r_{16}/\Lambda$	0.1	0.16	0.17	0.14	0.16	0.15	0.16	0.15	0.12	0.14	0.13	0.15	0.14	0.13	0.13	0.11	0.16	0.14	0.17	0.13	0.13
$r_{17}/\Lambda$	0.17	0.11	0.12	0.14	0.14	0.16	0.12	0.16	0.15	0.13	0.11	0.13	0.13	0.11	0.15	0.14	0.12	0.13	0.14	0.12	0.14
$r_{18}/\Lambda$	0.15	0.21	0.18	0.17	0.18	0.14	0.19	0.17	0.17	0.16	0.17	0.16	0.2	0.2	0.22	0.17	0.18	0.22	0.18	0.17	0.19
$r_{19}/\Lambda$	0.17	0.16	0.18	0.18	0.16	0.17	0.17	0.13	0.14	0.14	0.16	0.14	0.18	0.19	0.14	0.12	0.17	0.16	0.15	0.17	0.17
$r_{20}/\Lambda$	0.13	0.15	0.12	0.15	0.18	0.15	0.16	0.15	0.15	0.16	0.15	0.14	0.14	0.11	0.16	0.17	0.12	0.14	0.18	0.14	0.13
$h_{l1}/\Lambda$	1.58	1.74	1.71	1.78	1.63	1.89	1.61	1.75	1.78	1.6	1.64	1.73	1.67	1.57	1.82	1.63	1.88	1.91	1.71	1.66	1.54
$h_{l2}/\Lambda$	1.7	1.58	1.48	1.75	1.48	1.77	1.69	1.67	1.58	1.73	1.46	1.64	1.63	1.85	1.72	1.54	1.48	1.85	1.74	1.48	1.52
$l_{l1}/\Lambda$	0.94	0.79	0.81	0.8	0.77	0.79	0.78	0.87	0.83	0.9	0.86	0.85	0.85	0.95	0.81	0.83	0.88	0.96	0.93	0.78	0.92
$l_{l2}/\Lambda$	0.87	0.71	0.78	0.7	0.77	0.67	0.79	0.81	0.8	0.78	0.86	0.83	0.72	0.86	0.69	0.69	0.73	0.86	0.83	0.74	0.88
$h_{r1}/\Lambda$	1.98	1.82	1.69	1.89	1.84	1.79	1.73	1.86	1.94	1.96	1.79	1.72	1.73	1.98	1.67	1.92	1.63	2	1.57	1.8	1.84
$h_{r2}/\Lambda$	1.74	1.81	1.65	1.98	1.65	1.83	1.78	1.81	1.72	1.84	1.64	1.88	1.69	1.81	1.76	1.77	1.73	1.88	1.85	1.6	1.8
$l_{r1}/\Lambda$	0.88	0.84	0.95	0.81	0.9	0.98	0.95	0.87	0.86	0.87	0.79	0.9	0.94	0.81	0.89	0.91	0.96	0.87	0.85	0.88	0.84
$l_{r2}/\Lambda$	0.9	0.91	1	0.84	0.89	0.95	0.93	0.99	0.81	0.93	0.85	0.97	0.88	0.8	1	0.9	0.97	0.9	0.95	0.82	0.86
$d_{l}/\Lambda$	−3.7	−3.4	−3.5	−3.7	−3.4	−3.7	−3.4	−3.4	−3.5	−3.4	−3.2	−3.5	−3.4	−3.4	−3.6	−3.3	−3.6	−3.5	−3.5	−3.5	−3.6
$d_{r}/\Lambda$	3.7	3.5	3.5	3.4	3.7	3.8	3.6	3.8	3.4	3.6	3.7	3.7	3.7	3.7	3.6	3.6	3.6	3.9	3.7	3.5	3.5
Λ (μm)	5.1	5.2	4.8	4	5.1	4.2	5.1	4.6	5.1	5.1	5.1	5	5.1	5.2	5.8	5.2	4.5	4.5	4.1	5.1	5.7
EMA (μm${}^{2}$)	779	644	502	456	642	498	617	635	705	660	650	601	710	604	**880**	702	493	494	428	605	656
$L_{FX}$ (dB/m)	0.52	0.13	0.54	0.13	0.27	0.24	0.29	0.5	0.45	0.23	0.41	0.55	0.27	0.14	0.23	0.42	0.43	0.26	**0.12**	0.44	0.18
$L_{FY}/L_{FX}$	3.72	3.85	2.38	4.29	2.39	2.05	3.37	3.52	3.39	3.91	3.28	3.74	3.16	3.71	**4.95**	3.87	3.35	4.29	4.47	3.24	4.68
$L_{H}$ (dB/m)	59	57	364	190	67	241	119	115	79	85	113	241	59	57	16	33	**485**	396	233	181	52
$L_{FY}$ (dB/m)	1.92	0.49	1.28	0.54	0.64	0.5	0.98	1.77	1.54	0.92	1.34	**2.05**	0.85	0.51	1.12	1.64	1.44	1.14	0.52	1.43	0.85
$M^{2}$	1.24	1.16	1.14	1.06	1.16	1.06	1.17	1.16	1.23	1.15	1.19	1.15	1.20	1.15	1.34	1.27	1.09	1.10	**1.05**	1.18	1.32
$|M_{x}^{2}-M_{y}^{2}|\;\times \;10^{3}$	110	26	5	14	6	33	17	111	6	20	18	14	18	73	3	4	14	21	15	8	66
